# Coats disease in adolescence and adulthood with preserved vision after laser photocoagulation monotherapy: two case reports

**DOI:** 10.1186/s13256-022-03474-9

**Published:** 2022-07-18

**Authors:** Gitalisa Andayani Adriono, Andi Marsa Nadhira, Sausan Rasyid Mahfudz

**Affiliations:** 1 Department of Ophthalmology, Faculty of Medicine Universitas Indonesia, Dr. Cipto Mangunkusumo National General Hospital, Jakarta, Indonesia; 2 Jakarta Eye Center Clinics and Hospitals, Jakarta, Indonesia; 3 Faculty of Medicine Universitas Indonesia, Dr. Cipto Mangunkusumo National General Hospital, Jakarta, Indonesia

**Keywords:** Coats disease, Adolescent, Adult, Laser photocoagulation, Preserved visual acuity

## Abstract

**Background:**

This case report describes two rare cases of Coats disease in nonjuvenile patients with preserved vision.

**Case presentation:**

Two otherwise healthy Asian males aged 15 and 29 years old presented with unilateral gradual blurred vision and scotoma, respectively. Visual acuity was 6/6 (0 logMAR) with no other abnormalities observed in the anterior segment of the eyes. Both posterior segment examinations and fluorescein angiography revealed retinal telangiectatic vessels, exudation, and hemorrhage. Additionally, optical coherence tomography of the first patient showed subfoveal fluid. Both patients were diagnosed with stage 2 Coats disease. Laser photocoagulation was performed on both patients. The first patient showed initial good response to therapy with resolution of the subfoveal fluid; however, he developed cataract and underwent cataract surgery after 3 years. In the second case, although the exudates and hemorrhage still persisted, the macula was spared and the patient did not have visual complaints. No adverse events were reported, and final visual acuity of both patients remained 6/6.

**Conclusions:**

Coats disease in adolescence and adulthood may present with good vision. In mild and moderate cases of Coats disease, laser photocoagulation monotherapy may help preserve vision. Nevertheless, as recurrences and complications may still occur later in life, lifelong monitoring is recommended.

## Background

Coats disease is an idiopathic disorder that has retinal exudation, telangiectasia, and aneurysms as the hallmarks of the condition [1, 2]. This disease is usually unilateral with no racial predilection [3]. It generally affects males and is usually diagnosed at early age, with average age at diagnosis being 6 years [2]. In some cases, Coats disease may also be diagnosed in late childhood and adulthood, with features resembling the juvenile form, although it is usually localized, limited, and progressing at a slower rate than it does in children [4, 5]. Management varies from ablative therapy to surgery. In childhood cases of Coats disease, the visual prognosis is generally poor, with the majority presenting visual acuity (VA) of 20/200 (1.00 logMAR) or worse. However, in late childhood or adolescent and adult cases, the presenting vision may be better, with the majority of patients reaching a stable final VA. [6] This report describes the clinical course in two relatively rare cases of Coats disease, diagnosed in an adolescent and an adult patient. Good vision was preserved in both patients after laser photocoagulation monotherapy.

## Case presentation

### Case 1

**Fig. 1:**
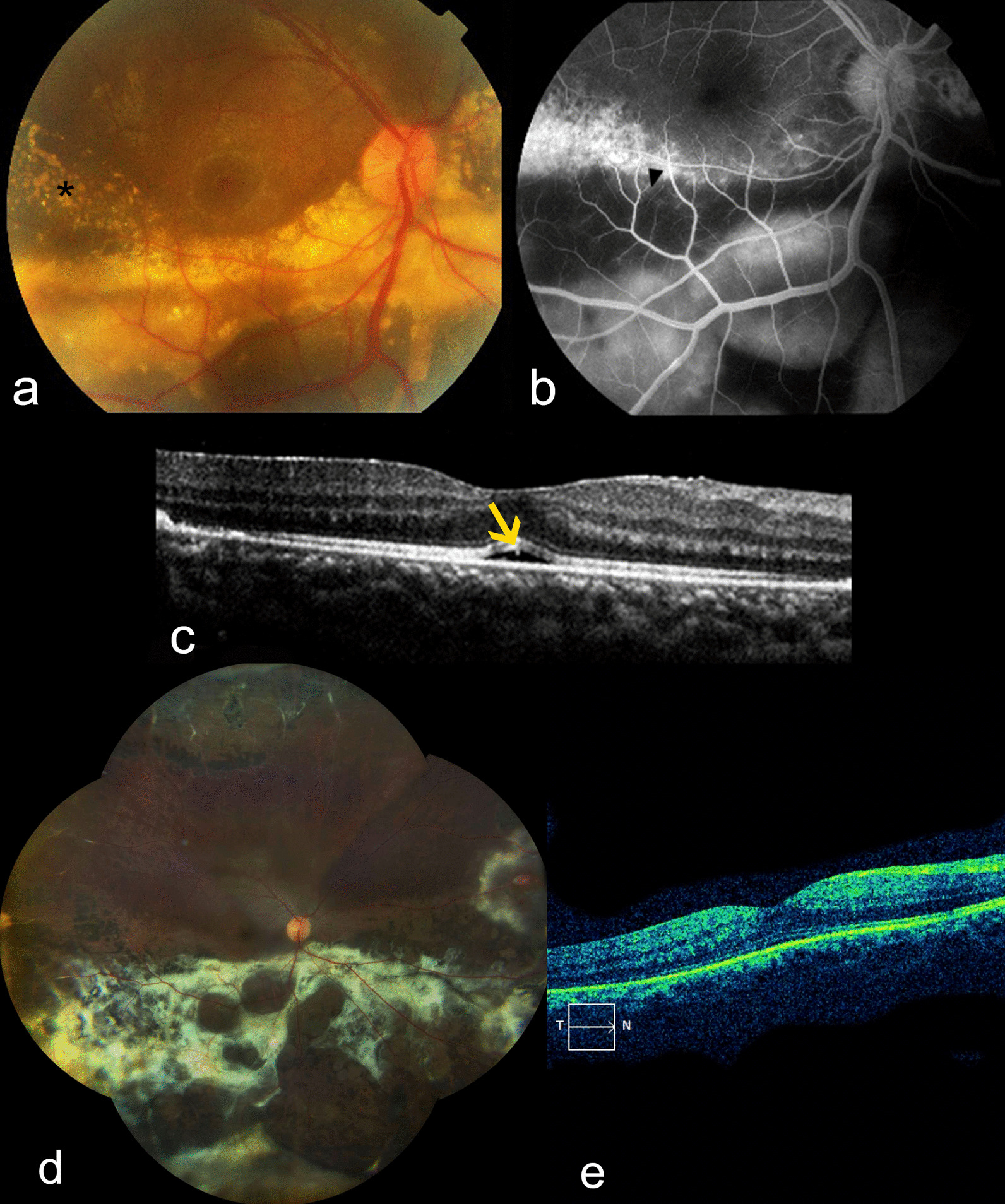
Fundus photography (**a**) and FA (**b**) of the right eye, which show lipid exudates in the retina of the right eye (asterisk) and telangiectatic vessels (black arrowhead). Optical coherence tomography (**c**) showing subfoveal fluid on the right eye (yellow arrow). Vision of both eyes was 6/6 (0 logMAR). Retinal and laser scars with subretinal fibrosis of the right eye were found upon posterior segment examination after laser photocoagulation (**d**). Optical coherence tomography (**e**) showing normal fovea

A 15-year-old Asian male was referred with gradual blurred vision of the right eye for 1 month. No other vision-related complaints were experienced. The patient had no previous history of systemic and ocular diseases. He was previously diagnosed with posterior uveitis of the right eye. His best corrected visual acuity (BCVA) was 6/6 (0 logMAR) on both eyes, with minus cylindrical lens 1.25 diopters axis 5° and minus cylindrical lens 0.25 diopters axis 5° on the right and left eye, respectively. Intraocular pressure (IOP) and eye movement were within normal limits. No signs of inflammation and cataract were observed in the anterior segment of either eye. Posterior segment examination on his right eye revealed extensive lipid exudates, especially in the inferior retina, sparing the foveal area (Fig. 1a). Fluorescein angiography (FA) was performed, showing hyperfluorescence indicating telangiectatic vessels, leakages corresponding to areas of lipid exudation, and hypofluorescence indicating capillary nonperfusion (CNP), predominantly within the inferior retina. Macula was normal (Fig. 1b). Despite the normal macula upon fundus photo and FA, optical coherence tomography (OCT) of the right eye revealed a small amount of subfoveal fluid (Fig. 1c).

Patient was then diagnosed with stage 2 Coats disease. After 2 weeks of observation and treatment with oral and topical nonsteroidal anti-inflammatory drugs (NSAID), there was no improvement in subjective complaints and clinical examination, showing persistent extensive retinal lipid exudation of right eye. The patient then underwent scatter laser photocoagulation on his right eye. On follow-up 2 months later, retinal fibrous exudates and laser scars were found on posterior segment examination. His right eye vision remained 6/6 (0 logMAR).

The patient did not come for follow-ups until 3 years later, when he was already 19 years old. He complained of decreased vision of the right eye for the last few months. Examinations revealed right eye BCVA of 6/10 (0.2 logMAR) (with minus spherical lens 2.00 diopters) and left eye 6/6 (0 logMAR) (with minus cylindrical lens 0.25 diopters axis 20°), and posterior subcapsular cataract of the right eye. Retinal and laser scars were found mainly in the inferior retina (Fig. 1d), and the fovea area looked normal on OCT, with disappearance of the initial subfoveal fluid (Fig. 1e). Cataract surgery was done on his right eye. One month after surgery, his right eye vision improved to 6/6 (0 logMAR).

### Case 2

**Fig. 2:**
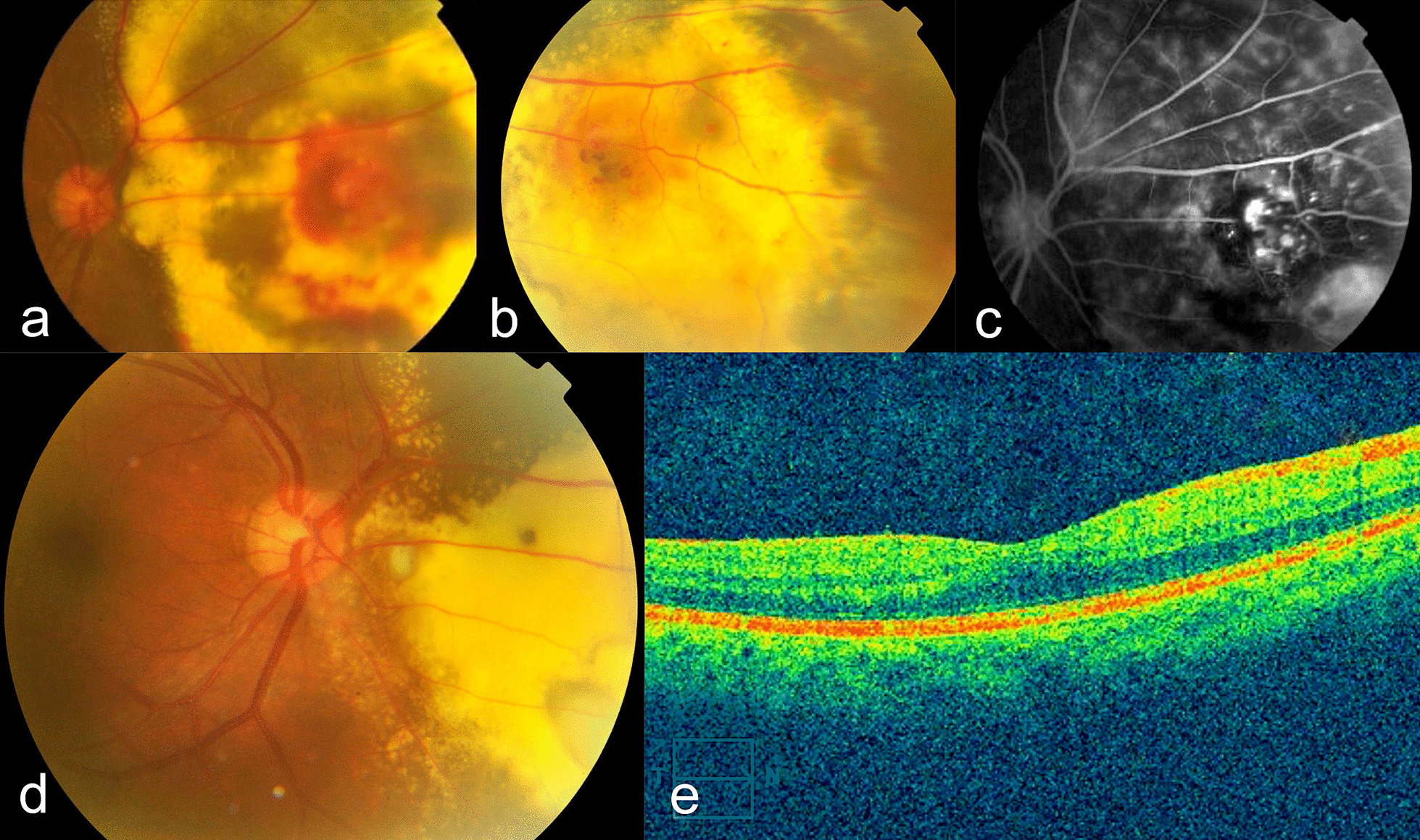
Fundus photo of right eye upon first examination, showing extensive exudate and hemorrhage (**a**) and (**b**) in the nasal retina, and FA showing telangiectatic vessels and multiple area of leakage, blocking from intraretinal hemorrhage, as well as areas of capillary nonperfusion (**c**). Fundus photo of the right eye (**d**) after laser photocoagulation showed remaining exudates and hemorrhage despite good VA, while optical coherence tomography (**e**) revealed normal fovea

A 29-year-old Asian male came to the clinic with a chief complaint of a shadow in the left side of his right eye for the past 2 months. He was once diagnosed with posterior uveitis, with differential diagnosis of Coats disease. He was tested positive for *Mycobacterium tuberculosis* (MT) and Interferon-Gamma Release Assay (IGRA). History of previous medications included high dose of oral methylprednisolone. Anterior segment, IOP, and eye movement were normal on both eyes. Fundus examination revealed retinal exudation and telangiectatic vessels, with presence of intraretinal hemorrhage, located in the nasal region of the right fundus (Fig. 2a and b). Temporal, superior, and inferior areas of macula were within normal limits. BCVA was 6/6 (0 logMAR) on both eyes. FA showed hyperfluorescence from telangiectasia and leakage, and hypofluorescence due to blocking in the hemorrhagic area, and capillary nonperfusion (Fig. 2c).

Diagnosis of stage 2 Coats disease was established. The patient was then treated with laser scatter and focal/grid photocoagulation in nasal area. Three months after laser photocoagulation, exudates and hemorrhage were still present; however, BCVA of the right eye remained 6/6 (0 logMAR) (with minus spherical lens 1.00 diopters and minus cylindrical lens 0.25 diopters axis 110°). No adverse events were reported. This patient relocated to another province, and was monitored through e-mail, which revealed that he currently had no visual complaints on his treated eye.

## Discussion

Coats disease is a disease that is associated with retinal exudation, and telangiectatic and aneurysmal retinal vessels [1, 2]. Coats disease can be diagnosed in infancy, late childhood or adolescence, and adulthood [2, 5]. Both of our patients were male, with symptoms first presenting later in adolescence (case 1) and adulthood (case 2).

No etiology has been established and no systemic conditions are associated with Coats disease. A few possible causes have been proposed, such as a genetic cause, relation to other exudative retinopathies with similar ocular phenotype, and vascular endothelial growth (VEGF) immunoreactivity [7, 8]. There are two mechanisms that cause retinal damage in Coats disease. First, endothelial breakdown of blood-retinal barrier results in plasma leakage, eventually impairing the vessels. Secondly, the presence of abnormal pericytes and endothelial cells in retinal blood vessels leads to formation of abnormal retinal vasculature and aneurysms [9]. These mechanisms cause exudates to leak into the retina, leading to retinal thickening, cyst formation, and retinal detachment [9]. Following exudate resolution, subretinal pigmentation and fibrosis usually take place. This may hinder visual recovery, especially if the pathologies are located in the fovea [1].

In adults, painless vision loss is the most common chief complaint, while child patients usually present with more variable symptoms, such as poor vision, nystagmus, strabismus, and leukocoria [2, 4, 5]. Coats disease is mainly unilateral, but if bilateral involvement is suspected, the features in one eye are usually more intense than in the other. Therefore, other causes of bilateral exudative retinopathy should also be excluded [3, 4]. These two cases were first referred with the diagnoses of posterior uveitis. Although the second patient had positive results related to tuberculosis, the first patient reported no systemic risk factors. This is common in clinical practice, as it has been reported that more than 50% of Coats disease cases are referred as other diseases, including retinal and choroidal inflammatory disorders [3].

Visual acuity in Coats disease usually ranges from normal vision to no light perception, depending on the severity of the disease and macular involvement. In our cases, despite the fact that visual symptoms were present, which we understood as scotomas corresponding with the pathologic lesions, initial visions were good, with both patients showing VA of 6/6 (0 logMAR). This finding is quite rare, even as the majority of older patients with Coats disease reportedly present with better vision compared with child patients. Kang *et al*., reported that visual acuities of patients with Coats disease at presentation were similar in both child and adult groups, with mean visual acuity of only 20/162 (0.9 logMAR) [10]. A cohort study by Dalvin *et al*., consisting of large samples of patients with Coats disease, found that younger age (3 years old and younger) at diagnosis is linked to more advanced stage, worse visual prognosis, and higher chance of enucleation [6].

Retinal telangiectasia, often referred to as “light bulb” telangiectasia, is the characteristic lesions predominantly found in temporal macula and mid-periphery [3, 10]. Other findings may include subretinal and/or intraretinal exudation, which is composed of cholesterol crystals, most commonly located in the macular area [1, 3]. Unless hemorrhage, retinal breaks, and neovascularization occur, vitreous is usually clear [3]. In advanced cases, exudative retinal detachment may develop. Shields classified Coats disease into five stages: stage 1, in which only retinal telangiectasia is found; stage 2, where telangiectasia and exudates are observed; stage 3, in which exudative retinal detachment develops; stage 4, where total retinal detachment and glaucoma are encountered; and stage 5, where the disease has advanced to end stage, occasionally with phthisis bulbi [1, 2]. Both of our patients were categorized as stage 2 because no retinal detachment was observed. This is concordant with previous studies reporting that nonjuvenile cases are usually diagnosed at stage 2 [2]. Despite having similar clinical features with juvenile cases, adult patients with Coats disease generally involve smaller retinal area and progress less rapidly [4–6, 10].

Excellent central vision in these two cases was preserved owing to the location of the lesions, where the macula was spared or not significantly affected. In the first case, the retinal exudation and hemorrhage involved only the inferior half of the retina, whereas in the second case, the pathology involved the nasal part of the retina. Kang *et al*. found that the childhood group of patients with Coats disease had more frequent macular exudation and significantly diffuse ring exudation [10]. Most patients with Coats disease usually have normal anterior segment; however, findings like cataract, rubeosis iridis, shallow anterior angle, anterior chamber cholesterolosis, megalocornea, and corneal edema may be observed in some cases [2, 3].

Other than ophthalmoscopy, several ancillary tests can be utilized to help distinguish Coats disease from other entities, and to monitor the disease progression. FA detects vascular anomalies, such as micro- and macro-aneurysms, areas of telangiectasias, beading of vessel walls, and vascular communicating channels [2, 3]. OCT helps determine retinal thickening and monitor macular edema [2, 10]. We performed both FA and OCT on both patients. FA in both cases showed telangiectatic vessels and areas of leakage, while OCT revealed mild subfoveal fluid on our first case upon initial examination (despite normal VA), which subsequently disappeared during follow-up.

Treatment of Coats disease aims to improve vision and preserve ocular anatomy [1, 11]. Various treatment modalities are available, and the choices depend on the disease severity and surgeon’s preference. Treatment includes cryotherapy, laser photocoagulation, external drainage of subretinal fluid, scleral buckling, and pars plana vitrectomy (PPV). Adjunctive intravitreal injection of corticosteroids and anti-VEGF has also been used in cases with subretinal exudates and macular edema [2, 12]. Patients with only retinal telangiectasia may be observed vigilantly, but if the disease progresses, treatment needs to be instigated [1]. As shown in our cases, in mild to moderate Coats disease where telangiectasia and exudates are present with no or mild retinal detachment, laser photocoagulation and cryotherapy are recommended [1]. In advanced stages where subtotal or total retinal detachment and glaucoma are present, PPV is usually required [2]. Enucleation may be indicated for end-stage disease with painful eye, while nonpainful eye can be observed without any treatment [9, 11, 13].

Laser photocoagulation for Coats disease is usually guided by FA, with the laser applied to obliterate defective vessels in order to stop further leakage and initiate exudate resorption [1]. Cryotherapy may be suitable in cases with peripheral lesions, subretinal fluid, or thick exudation; however, laser photocoagulation is still preferred owing to the possible transient increase in subretinal exudation and transient retinal detachment after cryotherapy [1]. We decided to treat the patients using laser photocoagulation only, with the aim to treat leaking telangiectasias and promote resorption of subretinal fluid and exudates. Furthermore, because both our patients showed mild to moderate features with little to no macular involvement, we were quite confident in choosing laser photocoagulation monotherapy. We applied focal, FA-guided laser photocoagulation in telangiectatic and nonperfusion areas, leaving healthy tissues intact. Usually, more than one treatment session of laser photocoagulation may be required, especially if more than two quadrants of the eye are involved and if exudation persists after laser treatment [14]. It is recommended to set the follow-up sessions 1–3 months apart from the previous one, to allow time for exudate resolution and monitor the possible complication of laser [1].

The first patient responded well to the treatment with no adverse events, and the initial subretinal fluid resolved after laser photocoagulation. On final follow-up, 3 years after laser treatment, retina was stable, showing fibrosis and scarring without active exudation. However, in the second case, there was still remaining exudation and hemorrhage 3 months after laser treatment. This may warrant the need for further investigations, including repeat retinal diagnostic imaging and systemic work-up to exclude other confounding diseases (as the patient tested positive for tuberculosis). The patient may also require further treatment with multimodalities such as additional laser, intravitreal, or periocular triamcinolone, or intravitreal anti-VEGF injections.

The prognosis of Coats disease depends on the severity of the disease. Presence of macular edema, epiretinal membrane, optic atrophy, and subfoveal fluid, as well as exudation, fibrosis, and hemorrhage, contributes to poor vision [2]. Recurrences may also occur many years later, marked by reappearance of exudates after seemingly successful resolution of the disease. Cataract may occur as a secondary complication of Coats disease. In our first case, the patient later developed cataract, which required surgery. Therefore, owing to possible recurrences and complications, we recommended close monitoring of patients even after the disease has stabilized. Patients are usually advised to have lifelong follow-up visits, preferably once every 6 months [15].

## Conclusion

Coats disease is an idiopathic, progressive disease that predominantly affects male children, but may also present later in adolescents and adults. Our report shows that, in rare cases found in older patients, the visual prognosis is favorable. Despite many options for treatment, mild and moderate cases with sparing of the macula may only need sectoral or FA-guided laser photocoagulation, which can preserve vision. Lifelong follow-up is recommended to monitor for recurrences, complications, and conditions requiring further treatment.

## Data Availability

Not applicable.

## Abbreviations

VA: Visual acuity
BCVA: Best corrected visual acuity
IOP: Intraocular pressure
FA: Fluorescein angiography
OCT: Optical coherence tomography
NSAID: Nonsteroidal anti-inflammatory drugs
MT: *Mycobacterium tuberculosis*
IGRA: Interferon-Gamma Release Assay
VEGF: Vascular endothelial growth
PPV: Pars plana vitrectomy
